# Synthesis and Antimicrobial Activity of Some Novel Cross-Linked Chitosan Hydrogels

**DOI:** 10.3390/ijms130911194

**Published:** 2012-09-10

**Authors:** Nadia Ahmed Mohamed, Mona Mohamed Fahmy

**Affiliations:** Department of Chemistry, Faculty of Science, Cairo University, Giza 12613, Egypt; E-Mail: m.fahmy17@yahoo.com

**Keywords:** chitosan, chemical cross-linking, hydrogels, antimicrobial activity

## Abstract

Four novel hydrogels based on chitosan were synthesized via a cross-linking reaction of chitosan with different concentrations of oxalyl bis 4-(2,5-dioxo-2*H*-pyrrol- 1(5*H*)-yl)benzamide. Their structures were confirmed by fourier transform infrared X-ray (FTIR), scanning electron microscopy (SEM) and X-ray diffraction. The antimicrobial activities of the hydrogels against two crop-threatening pathogenic fungi namely: *Aspergillus fumigatus* (*A. fumigatus*, RCMBA 06002), and *Aspergillus nige*r (*A. niger*, RCMBA 06106), and five bacterial species namely: *Bacillis subtilis* (*B. subtilis*, RCMBA 6005), *Staphylococcus aureus* (*S. aureus*, RCMBA 2004), *Streptococcus pneumoniae* (*S. pneumonia*, RCMB 000101) as Gram positive bacteria, and *Salmonella typhimurium* (*S. typhimurium*, RCMB 000104), and *Escherichia coli* (*E. coli*, RCMBA 5003) as Gram negative bacteria have been investigated. The prepared hydrogels showed much higher antimicrobial activities than that of the parent chitosan. The hydrogels were more potent in case of Gram-positive bacteria than Gram-negative bacteria. Increasing the degree of cross-linking in the hydrogels resulted in a weaker antimicrobial activity.

## 1. Introduction

Hydrogels are cross-linked hydrophilic polymer networks that are insoluble but capable to absorb large amounts of water or biological fluids and drastically increase in volume [1]. They are usually formed by the covalent cross-linking of linear polymers to form a network [2]. Heterogeneous polymer mixtures may also be used to form hydrogels without the need for covalent cross-linking [3]. It is well known that the physicochemical properties of the hydrogel depend not only on the molecular structure, the gel structure, and the degree of cross-linking, but also on the content and state of the water in the hydrogel [4]. Polymer gels have been studied for their applications in a variety of fields, such as chemical engineering, foodstuffs, agriculture, medicine, and pharmaceuticals for controlled drug delivery systems. They may also have applications as muscle-like soft linear actuators, robotics, sensors, biomimetic energy transducing devices, and separation techniques [5–7]. It is well established that polysaccharides possess better biocompatibility, biodegradability, non-toxicity, and easily modified ability than various synthetic polymers. In response to the environmental concern, considerable research has been recently directed towards the use of naturally abundant polymers such as chitin, cellulose, chitosan and their derivatives for production of hydrogels. Chitosan is derived from chitin, by a deacetylation with strong alkali. It is actually a copolymer of glucosamine and *N*-acetyl glucosamine. It has many properties that have generated interest in its use such as water binding capacity, biodegradability, biocompatibility, non-toxicity, bioactivity, metal uptake capacity, fat binding capacity and antimicrobial activity [5–7]. It can be also used as biological adhesive for its hydrogel forming ability [5,6]. In view of the structural similarities between chitosan and the glycosaminoglycans found in the extracellular matrix of mammalian tissue, many possible uses of chitosan-based hydrogels as scaffolds for tissue engineered medical products could be realized. Chemical cross-linking of chitosan is a highly versatile method to improve its physical and mechanical properties. To date, the most common cross-linkers used for fabrication of chitosan hydrogels are gluteraldehyde [8], formaldehyde [9], glyoxal [10], dialdehyde starch [11], epoxy compound [12], diethyl squarate [13], pyromellitic dianhydride [14], genipin [15], quinone [16] and diisocyanate [17–20]. On the other hand, some efforts have been made to prepare peptide chitosans as antioxidant and antimutagenic agents [21], as chelating agents [22] and as drug delivery systems [23,24].

Thus, it became of interest to synthesize four novel chitosan hydrogels containing peptide linkages using a new heterocyclic compound, oxalyl bis 4-(2,5-dioxo-2*H*-pyrrol-1(5*H*)-yl)benzamide, as a cross-linker. The cross-linking reaction may occur through ring opening of the maleimide moieties of the cross-linker with the amino groups of chitosan. The incorporation of the amide linkages of the cross-linker into chitosan may act as centers for bonding bioactive materials for drug delivery systems. In addition, the carbonyl and NH groups can act as centers for antimicrobial activity. The hydrogels will be characterized by fourier transform infrared (FTIR), scanning electron microscopy (SEM), X-ray diffraction (XRD), solubility and swell ability in various solvents. The antimicrobial activities of these hydrogels against two crop-threatening pathogenic fungi namely: *Aspergillus fumigatus* (*A. fumigatus*, RCMBA 06002), and *Aspergillus nige*r (*A. niger*, RCMBA 06106), and against five bacterial species namely: *Bacillis subtilis* (*B. subtilis*, RCMBA 6005), *Staphylococcus aureus* (*S. aureus*, RCMBA 2004), *Streptococcus pneumoniae* (*S. pneumonia*, RCMB 000101) as Gram positive bacteria*, Salmonella typhimurium* (*S. typhimurium*, RCMB 000104), and *Escherichia coli* (*E. coli*, RCMBA 5003) as Gram negative bacteria are also described. The effect of the cross-linker content on the hydrogel characteristics will be reported.

## 2. Results and Discussion

**Scheme 1:**
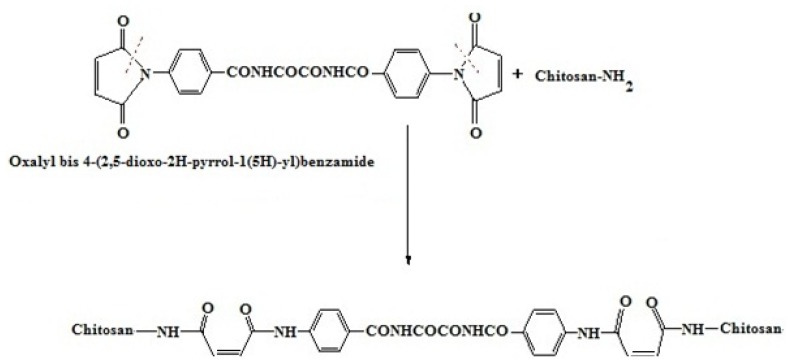
Synthesis of chitosan hydrogels (H_0.5_–H_5_).

In the present work, oxalyl bis 4-(2,5-dioxo-2*H*-pyrrol-1(5*H*)-yl)benzamide was used as a cross-linking agent to modify chitosan. Chitosan (10 mmol) was reacted with four different concentrations of the cross-linker (0.5, 1.0, 2.5, and 5 mmol) via ring opening of its maleimide moieties with the amino groups of chitosan to give four cross-linked chitosan hydrogels designated as H_0.5_, H_1.0_, H_2.5_, and H_5_ of increasing cross-linking degree, respectively (Scheme 1).

### 2.1. FTIR Characterization of the Hydrogels

**Figure 1:**
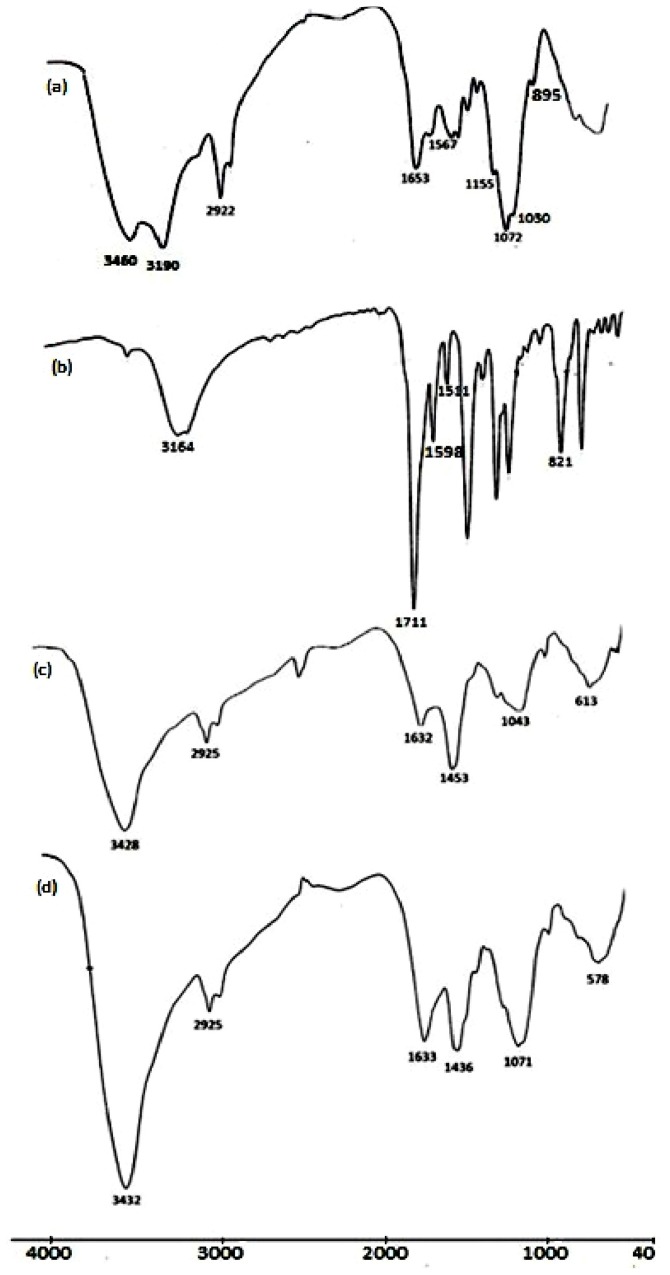
Fourier transform infrared FTIR spectra of chitosan and its hydrogels: (**a**) Chitosan; (**b**) oxalyl bis 4-(2,5-dioxo-2*H*-pyrrol-1(5*H*)-yl)benzamide; (**c**) H_0.5_; (**d**) H_5_.

An additional proof for the synthesis of the hydrogels is given by their FTIR spectra as shown in Figure 1. The FTIR spectrum of chitosan (Figure 1a) showed four strong peaks at 1155, 1072, 1030, and 895 cm^−1^, which were characteristic peaks of the saccharide structure. The very strong broad peak around 3600 to 3000 cm^−1^ should be assigned to the stretching vibration of O–H, the extension vibration of the N–H, and the intermolecular hydrogen bonds of the polysaccharide. Primary amines have two peaks in this region at 3460 and 3190 cm^−1^. There were weak absorption peaks at 1653 and 1567 cm^−1^ corresponded to amide I and amide II, respectively, which indicated that chitosan had a high deacetylation degree. On comparing the FTIR spectra of chitosan (Figure 1a) and its hydrogels H_0.5_ and H_5_ (Figure 1c,d), it was found firstly that the broad band between 3600 and 3000 cm^−1^ due to the O–H and N–H group stretching vibration was observed. In addition, the characteristic doublet peak of the NH_2_ group at 3460 and 3190 cm^−1^ disappeared and a single peak around 3428 cm^−1^ for the NH group appeared. These results support that the NH_2_ group had reacted with the cross-linker. Further, the very strong characteristic peak of the C=O group of the imide ring of the cross-linker at 1711 cm^−1^ (Figure 1b) disappeared upon cross-linking with chitosan. This suggests that the cross-linking reaction occurs via imide ring opening. Secondly, a new peak around 1632 cm^−1^ appeared in the spectra of the hydrogels, which is the characteristic band for the C=O group of the amide linkage overlapped with both the C=C (aliphatic), C=C (aromatic) groups. Its intensity increased with increasing amount of the cross-linker incorporated into the hydrogels. This evidence confirms the structure of the prepared hydrogels.

### 2.2. Scanning Electron Microscopy Observations of the Hydrogels

**Figure 2:**
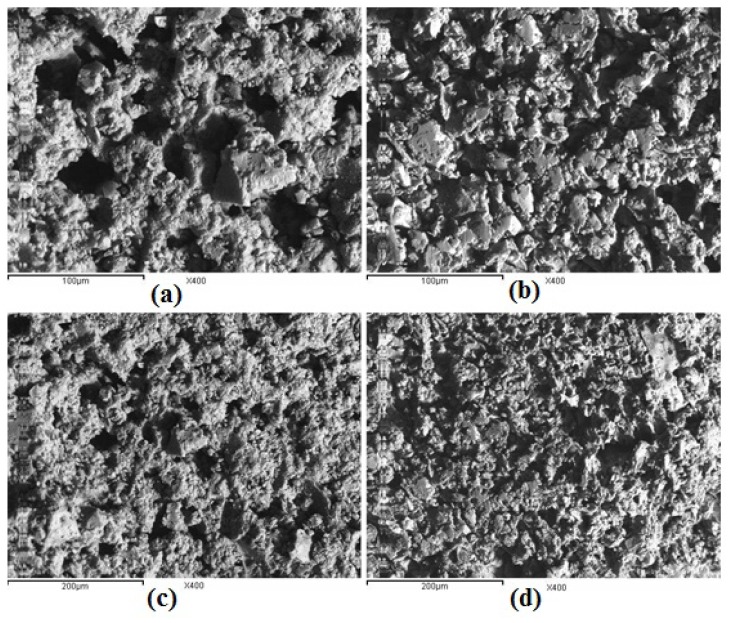
Scanning electron micrographs of the hydrogels surfaces at a magnification of 400×: (**a**) H_0.5_; (**b**) H_1_; (**c**) H_2.5_; (**d**) H_5_.

Microstructures of the hydrogels surface were investigated by scanning electron microscopy as presented in Figure 2. It could be seen that the hydrogels have a channel-like surface with an extremely porous structure. While the hydrogels have a similar surface appearance, the distribution and the size of their pores are different. The distribution of porosity became more uniform and dense with increasing concentrations of cross-linking moieties incorporated into the gel (H_5_ hydrogel). On the other hand, the pore size of the hydrogels decreased with the increase of the cross-linking density of the hydrogels, e.g., from H_0.5_ to H_5_ hydrogels.

### 2.3. X-ray Diffraction of TTUCS Hydrogels

**Figure 3:**
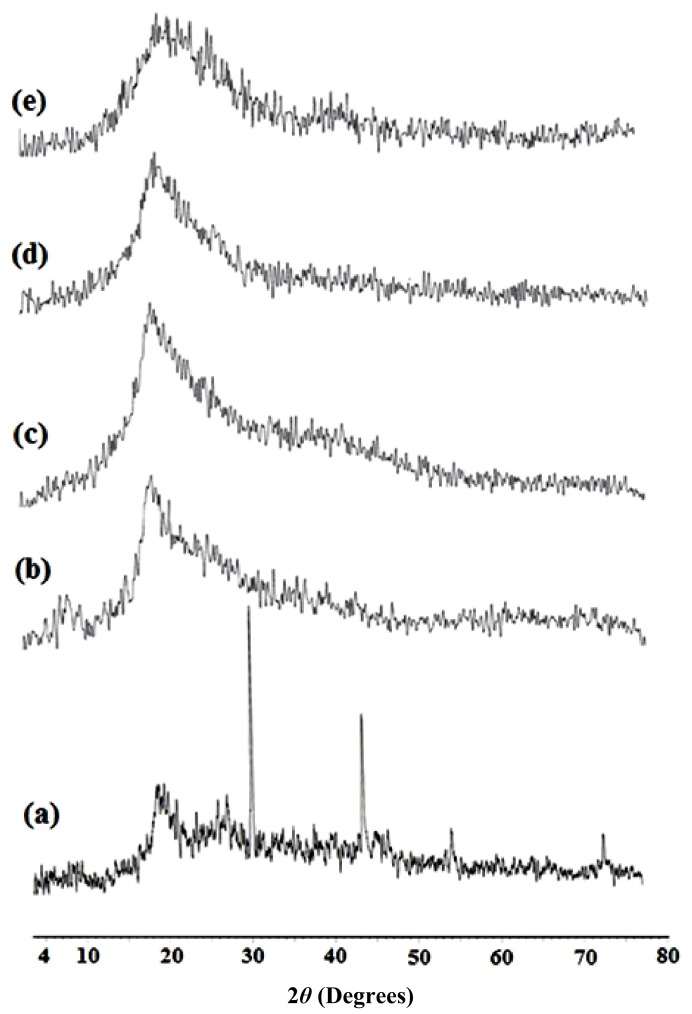
Powder X-ray diffraction of chitosan and its hydrogels: (**a**) Chitosan; (**b**) H_0.5_; (**c**) H_1_; (**d**) H_2.5_; (**e**) H_5_.

X-ray diffraction was employed to study the effect of cross-linking on the hydrogel morphology, and the powder X-ray diffractograms of chitosan and its hydrogels are presented in Figure 3a–e. From Figure 3a it can be seen that the two peaks showing the maximum intensity were obtained at 2*θ* = 31.6° and 2*θ* = 45.4°, indicating that chitosan is highly crystalline in nature. The incorporation of the cross-linker into chitosan severely decreases the intensity of both the peaks; that is, almost no peaks are obtained, which is clearly visible in Figure 3b–e. The cross-linked chitosan hydrogels showed an almost amorphous nature since the functional groups of chitosan underwent significant change after cross-linking. This suggested that a large number of hydrogen bonds in the chitosan powder was destroyed after crosslinking via NH_2_ groups, which efficiently destroyed the regularity of the packing of the original chitosan chains and resulted in the formation of amorphous hydrogels.

### 2.4. Solubility and Solvent Uptake Capacity of the Hydrogels

It is well known that chitosan is insoluble in both water and common organic solvents, but is readily soluble in dilute acetic acid solution to yield a hydrogel dissolved in an aqueous medium. Hence the solubility of the new hydrogels was studied in different solvents at room temperature. The results show that all the hydrogels are insoluble in acetic acid solution (1% *v*/*v*), DMF, DMSO, THF, NMP, chloroform, methylene chloride, acetone and methanol since no soluble fractions of all the hydrogels were obtained.

The results of swelling of the hydrogels in various solvents are summarized in Table 1. These results represent the average of three comparable experiments for each sample. The data showed that all the hydrogels are greatly swelled in all the investigated solvents, as they are much more hydrophilic in nature due to their highly polar amide linkages which can form many more hydrogen bonds with the investigated solvents. This indicates successful formation of cross-linked networks in these hydrogels. The highest degree of swelling for the hydrogels is observed with acetic acid solution (1% *v*/*v*) as compared with the aprotic solvents which may be attributed to the basicity of the amide linkages of the cross-linker. The highest swelling degree for the aprotic solvent is observed in DMSO due to its higher polarity. On increasing the cross-linking density, the swell ability decreases. This is well illustrated by the data reported for the swell ability in the case of H_5_ as compared with that of the other hydrogels irrespective of the nature of the investigated solvents.

### 2.5. The Antimicrobial Activity of the Hydrogels

Table 2 shows the antibacterial activity of the chitosan and its hydrogels using the inhibition zone method. Compared with chitosan, all the hydrogels have a higher antibacterial activity. Several mechanisms elucidating the antimicrobial activity of chitosan have been postulated. The most acceptable mechanism is the interaction between positively charged chitosan molecules and negatively charged microbial cell membranes. The interaction is mediated by the electrostatic forces between the protonated NH_3_^+^ groups of chitosan and the electronegative charges on the microbial cell surfaces. This electrostatic interaction results in a twofold interference: (i) by promoting changes in the properties of membrane wall permeability, thus provoking internal osmotic imbalances and consequently inhibiting the growth of microorganisms; and (ii) by the hydrolysis of the peptidoglycans in the microorganism wall, leading to the leakage of intracellular electrolytes such as potassium ions, and other low molecular weight proteinaceous constituents (e.g., protein, nucleic acid, glucose, and lactate dehydrgenase) [25]. Since such a mechanism is based on electrostatic interactions, it suggests that the greater the number of cationized groups, the higher the antimicrobial activity. The incorporation of hydrophilic cross-linker moieties into chitosan allowed the synthesis of hydrogels with higher hydrophilicity, with better swell ability in aqueous media and organic solvents, and with greater positive charge density, where the C=O and NH groups in hydrogels could be protonated and consequently the net positive charge was strengthened, leading to a better antibacterial activity. Another proposed mechanism is the binding of chitosan with microbial DNA, which leads to the inhibition of the mRNA and protein synthesis via penetration of chitosan into the nuclei of the microorganisms [26]. The cross-linker moieties incorporated onto hydrophilic chitosan part the chitosan chains away from each other, decrease their intermolecular hydrogen bonds, and increase their solubility; this is the reason for the ease of penetration of the hydrogels into the cells of microorganisms, thereby preventing the growth of the cell by preventing the transformation of DNA to RNA to obtain a higher antibacterial activity. The third mechanism is the chelation of metals, suppression of spore elements and binding to nutrients essential to microbial growth [27]. It is well established that the amide linkages have excellent metal-binding capacities [28]. This explains the observed higher antibacterial activity of hydrogels relative to the parent chitosan.

Moreover, both the chitosan and its hydrogels were more active against the Gram-positive bacteria than against the Gram-negative bacteria (Table 2). The strongest H_0.5_ hydrogel caused an inhibition zone diameter for *B. subtilis*, *S. aureus*, and *S. pneumonia* of 22.4, 20.6, and 19.9 mm, respectively, which corresponds to 16.4, and 13.9 mm for *S. typhimurium* and *E. coli*, respectively. This may be attributed to their different cell walls. The cell wall of Gram-positive bacteria is fully composed of peptide polyglycogen. The peptidoglycan layer is composed of net works with plenty of pores, which allow foreign molecules to come into the cell without difficulty and allow more rapid absorption of ions into the cell. But the cell wall of the Gram-negative bacteria is made up of a thin membrane of peptide polyglycogen and an outer membrane constituted of lipopolysaccharide, lipoprotein and phospholipids. Because of the complicated bilayer cell structure, the outer membrane is a potential barrier against foreign molecules with high molecular weight. Therefore, the hydrogels have different effects on the two kinds of bacteria. An additional evidence for the greater activity of the hydrogels against Gram-positive bacteria than against Gram-negative bacteria comes from their minimum inhibitory concentration (MIC) values. MIC is defined as the lowest concentration of an antimicrobial that will inhibit the visible growth of a microorganism after overnight incubation. The MIC values of the strongest antibacterial hydrogel (H_0.5_) are shown in Table 3. Since the MIC values of the H_0.5_ hydrogel against *B. subtilis*, *S. aureus*, and *S. pneumoniae* were 0.98, 3.91 and 3.91 μg/mL, its MIC values against *E. coli* and *S. typhimurium* were 125 and 62.5 μg/mL, respectively. Moreover, to attain a similar antibacterial activity, the MIC values of H_0.5_ hydrogel ranged from 1.5% to 3% of that of chitosan in the case of Gram positive bacteria and represented 25% of that of chitosan in the case of Gram negative bacteria.

It is interesting to note that the H_0.5_ hydrogel showed the highest antibacterial activity, relative to the other hydrogels, as judged by the highest inhibition zone diameter (Table 2). On the other hand, the H_5_ hydrogel exhibited the lowest antibacterial activity. The activities of the other hydrogels lie in between these two cases. Thus, the inhibitory effect decreased with increasing cross-linking density. The different inhibitory effect may be attributed to the extent of the swell ability of the hydrogels, which decreased with increasing cross-linker content incorporated into the hydrogels. The swell ability seems to improve the contact surface between the hydrogel and the bacteria. Also, it seems that the lower the degree of cross-linking of the hydrogels, the higher the degree of freedom of the cross-linked chitosan chains that can fulfill their antibacterial activity while still covalently being immobilized into the network.

The antifungal activities of the hydrogels against *A. fumigatus*, and *A. niger* are shown in Tables 4 and 5. The results show that all the hydrogels had effective activities against the tested fungi, compared with the parent chitosan, with inhibitory indices ranging from 12.4 to 20.1 mm inhibition zone (Table 4) and with MIC values of 3.91 up to 15.63 μg/mL for the strongest antifungal hydrogel H_0.5_ (Table 5). Further, for a comparable antifungal activity, the MIC values of H_0.5_ hydrogel ranged from 3% to 6% of that of chitosan. Generally chitosan has been reported as being very effective in inhibiting spore germination, germ tube elongation and radial growth [29]. The antifungal mechanism of chitosan involves cell wall morphogenesis with chitosan molecules interfering directly with fungal growth, similarly to the effects observed in bacteria cells [29]. The microscopic observation reported that chitosan molecules diffuse inside hyphae interfering with the enzyme activity responsible for fungus growth [30]. The higher swell ability of the hydrogels is expected to enhance the diffusion of the active ingredient inside the pathogens, which may lead to a disturbance of the enzyme activities responsible for the growth criteria, instead of the adsorption of the insoluble compounds on the fungal hyphae surface. The results also showed that the highest antifungal activity was observed for H_0.5_. This may be attributed to its higher swell ability. Pentachloronitrobenzene and chlorothalonil are usually used as fungicides; however, the chloro-groups in these fungicides constitute a big problem in the environment due to their toxicity [31]. The investigated hydrogels might be expected to induce lower pollution to the environment.

## 3. Experimental Section

### 3.1. Materials

Chitosan with a degree of deacetylation of 88% and a molecular weight of 2.0 × 10^5^ was purchased from Acros Organics, New Jersey, USA. All other chemicals and reagents were of analytical grade, from Aldrich and were used as received. The crop-threatening pathogenic fungi (*A. fumigatus*, RCMBA 06002; and *A. niger*, RCMBA 06106), and bacteria (*B. subtilis*, RCMBA 6005; *S. aureus*, RCMBA 2004; *S. pneumoniae*, RCMB 000101; *S. typhimurium*, RCMB 000104; and *E. coli*, RCMBA 5003) used for the antimicrobial assay were provided by the Reginol center for Mycology and Biotechnology Culture Collection.

### 3.2. Preparation of Oxalyl Bis 4-(2,5-dioxo-2*H*-pyrrol-1(5*H*)-yl)benzamide

**Scheme 2:**
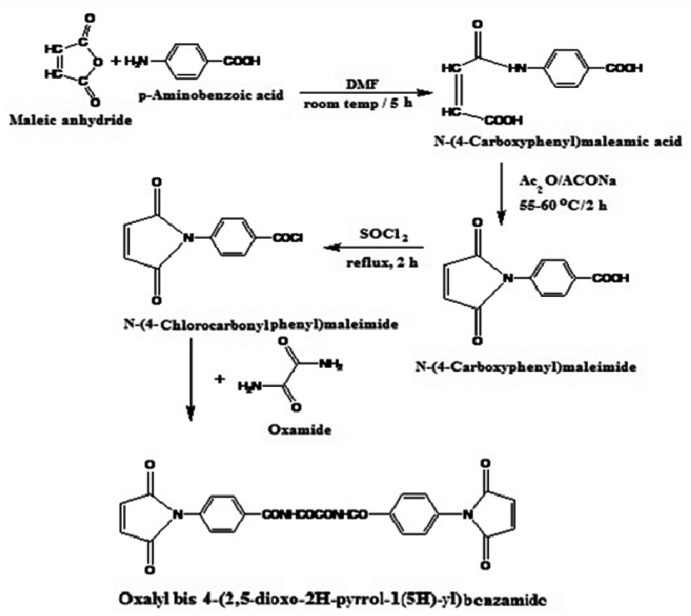
Synthesis of oxalyl bis 4-(2,5-dioxo-2*H*-pyrrol-1(5*H*)-yl)benzamide.

Oxalyl bis 4-(2,5-dioxo-2*H*-pyrrol-1(5*H*)-yl)benzamide was synthesized from maleic anhydride, *p*-amino-benzoic acid and oxamide, as shown in Scheme 2. Maleic anhydride (1 mol) and *p*-aminobenzoic acid (1 mol) were dissolved in DMF (320 mL), then the mixture was stirred at room temperature for 5 h under nitrogen atmosphere. The resulting solution was then poured into a large amount of water to precipitate crude *N*-(4-carboxyphenyl) maleamic acid, which was filtered, dried and recrystallized from water (yield = 97%; mp 223–225 °C) (Literature [32] mp 225–226 °C). Then, a mixture of *N*-(4-carboxyphenyl) maleamic acid (0.2 mol), acetic anhydride (100 mL) and anhydrous sodium acetate (2.5 g) was stirred at 55–60 °C for 2 h. The reaction mixture was poured onto a large amount of water to give crude *N*-(4-carboxyphenyl) maleimide, which was filtered and washed with water, dried and recrystallized from methanol:water (6:1) mixture (yield = 85%; mp 211–212 °C) (Literature [33] mp 208–210 °C). Then, a mixture of *N*-(4-carboxyphenyl)maleimide (0.16 mol), thionyl chloride (4.02 mol) and *tert*-butylcatechol (0.01 g) was refluxed for 2 h. Unreacted thionyl chloride was evaporated out, and then the residual product was recrystallized from benzene to obtain pure *N*-[4-(chlorocarbonyl)phenyl]maleimide, (yield = 73.3%; mp 166–167 °C) (Literature [32] mp 168–169 °C). Finally, a solution of 3.52 g (0.04 mol) oxamide dissolved in 100 mL DMF was stirred well, and allowed to cool at −10 °C using an ice-salt bath for 15 min. Then 18.84 g (0.08 mol) solid *N*-(4-chloro-carbonylphenyl) maleimide was added slowly with constant stirring for 1 h. The ice-salt bath was removed to let the temperature of the condensation reaction rise gradually to room temperature, and it was maintained for an additional 2 h with stirring. The reaction mixture was slowly poured onto methanol-water mixture (1:2), upon which a white precipitate of oxalyl bis 4-(2,5-dioxo- 2*H*-pyrrol-1(5*H*)-yl)benzamide was immediately formed. The product was filtered, dried and recrystallized from a methanol:water (1:1) mixture, (yield 75.8%; mp 233–235 °C). Elemental analyses: Calcd: 59.26% C; 2.88% H; 11.52% N; Found: 58.91% C; 2.96% H; 11.35% N. MS *m*/*z*: 486 (M^+^). FTIR spectrum (Figure 1b) showed a specific band for a maleimide moiety at 821 cm_−1_, while two strong bands appeared at 1511 and 1598 cm_−1_ corresponding to the stretching vibration for aromatic rings. A specific very strong band appeared at 1711 cm_−1_ for the C=O of the imide linkages of the maleimide moiety. Also the –NH– stretching band appeared at 3164 cm_−1_.

### 3.3. Synthesis of Cross-Linked Chitosan Hydrogels

Four different predetermined amounts of the cross-linker, oxalyl bis 4-(2,5-dioxo-2*H*-pyrrol-1(5*H*)- yl)benzamide, (0.5, 1.0, 2.5, and 5 mmol) were separately dissolved in 5 mL glacial acetic acid. Each solution was added to a chitosan solution (1.61 g, 10 mmol, in 100 mL of 1% acetic acid). The reaction mixture was stirred at 60 °C for 2 h, cooled, and the homogenous cross-linked hydrogels formed (Scheme 1) were neutralized with sodium bicarbonate solution to pH 7 to give yellowish white products which were submerged in methanol for 24 h for dewatering. The dewatered hydrogels were filtered and dried at 60 °C to constant weights to give the corresponding four new hydrogels designated as H_0.5_, H_1_, H_2.5_, and H_5_ of increasing degree of cross-linking.

### 3.4. Measurements

The reaction between chitosan and the cross-linker was confirmed using Tescan Shimadzu FTIR spectrophotometer (Model 8000, Japan). Hydrogel samples were ground well to make KBr pellets under hydraulic pressure of 400 kg/cm^2^ and spectra were recorded in the range of 400–4000 cm^−1^. In each scan, the amount of hydrogel sample and KBr were kept constant in order to know the changes in the intensities of the characteristic peaks with respect to the amount of the cross-linker.

Mass spectra were recorded on GCMS-QP 1000 ex spectra mass spectrometer operating at 70 eV.

Elemental analyses of the hydrogels were done in Perkin-Elmer (Model 2410 series II) C, H, N, S Analyzer (USA) at the Microanalytical Unit, Cairo University (Egypt).

Scanning electron microscopy observations of the hydrogels were carried out as follows: The dry samples spread on a double sided conducting adhesive tape, pasted on a metallic stub, were coated with a gold layer of 100 μm thickness using an ion sputter coating unit (Jeol S150A) for 2 min and observed with a Jeol-JXA-840A Electron Probe Microanalyzer. All the SEM photomicrographs were obtained using an accelerating voltage of 20 kV and at a magnification of 400×.

Powder X-ray diffraction patterns of the hydrogel samples were obtained using Brukur D8 Advance-Germany with a Ni monochromator. The power level was 40 kV/40 mA. The X-ray source was CuKα radiation. The samples were maintained stationary while scattering angles from 3° to 80° were scanned in the reflection mode at a scanning rate of 1° min^−1^.

Determination of the soluble fraction of the hydrogels: Weighed samples of each hydrogel were stirred overnight in 10 ml of each of the following solvents: acetic acid solution (1% *v*/*v*), DMF, DMSO, THF, NMP, chloroform, methylene chloride, acetone and methanol. The swollen samples were then dried in an oven at 60 °C to constant weights. The soluble fraction was calculated according to the following equation: Soluble fraction (%) = [(*W*_o_ − *W*_1_)/ *W*_o_] × 100, where *W*_o_, is the initial weight of the hydrogel and *W*_1_, is the weight of the oven dried hydrogel.

Determination of the swell ability of the hydrogels in various solvents: A known weight of the dry hydrogel sample was immersed in a single solvent (acetic acid solution (1% *v*/*v*), DMF, and DMSO) and kept undisturbed at room temperature until equilibrium swelling was reached. The swollen sample was then removed from the immersion media, quickly wiped with filter paper to remove the droplets on its surface and reweighed. The percent swelling was calculated using the following equation: Swelling (%) = [(*W*_1_ − *W*_o_)/ *W*_o_] × 100, where *W*_o_, is the weight of the dry hydrogel and *W*_1_, is the weight of the swollen hydrogel. Swelling measurements were made in triplicate, and the error was estimated to be within 1%.

Antibacterial activities were investigated using the agar well diffusion method. The activity of tested samples was studied against the *B. subtilis* (RCMBA 6005), *S. aureus* (RCMBA 2004), and *S. pneumoniae* (RCMB 000101) as Gram positive bacteria and *S. typhimurium* (RCMB 000104), and *E. coli* (RCMBA 5003) as Gram negative bacteria. Centrifuged pellets of bacteria from a 24 h old culture containing approximately 104–106 CFU (colony forming unit) per milliliter were spread on the surface of nutrient agar (typetone 1%, yeast extract 0.5%, NaCl 0.5%, agar 1%, 1000 mL of distilled water, pH 7.0) which was autoclaved under 121 °C for at least 20 min. Wells were created in medium with the help of sterile metallic bores and then cooled down to 45 °C. The activity was determined by measuring the diameter of the inhibition zone (in mm). One hundred microliters of the tested samples (10 mg/mL) were loaded into the wells of the plates. All compounds were prepared in DMSO, and DMSO was loaded as control. The plates were kept for incubation at 37 °C for 24 h and then the plates were examined for the formation of zone of inhibition. Each inhibition zone was measured three times by caliper to get an average value. The test was performed three times for each bacterium culture: penicillin and streptomycin were used as antibacterial standard drugs [34].

Antifungal activities were investigated by screening the tested samples separately in vitro against *A. fumigatus* (RCMBA 06002), and *A. niger* (RCMBA 06106) fungi on sabourad dextrose agar plates. The culture of fungi was purified by the single spore isolation technique. The antifungal activity was by agar well diffusion method [35] as follows:

Sabourad dextrose agar plates: A homogeneous mixture of glucose-pepton-agar (40:10:15) was sterilized by autoclaving at 121 °C for 20 min. The sterilized solution (25 mL) was poured into each sterilized Petri dish in laminar flow and left for 20 min to form the solidified sabourad dextrose agar plate. These plates were inverted and kept at 30 °C in an incubator to remove the moisture and to check for any contamination.

Antifungal assay: A fungal strain was grown in 5 mL sabourad dextrose broth (glucose:peptone; 40:10) for 3–4 days to achieve 105 CFU/mL cells. The fungal culture (0.1 mL) was spread out uniformly on the sabourad dextrose agar plates by sterilized triangular folded glass rod. Plates were left for 5–10 min so that the culture was properly adsorbed on the surface of sabourad dextrose agar plates.

Small wells (4 mm × 2 mm) were cut into the plates with the help of a well cutter and the bottom of the wells were sealed with 0.8% soft agar to prevent the flow of test sample at the bottom of the well. One hundred microliters of the tested samples (10 mg/mL) were loaded into the wells of the plates. All compounds were prepared in DMSO, and DMSO was loaded as control. The plates were examined for the formation of zone of inhibition. Each inhibition zone was performed three times for each fungus. Clotrimazole and itraconazole were used as antifungal standard drugs.

To determine the minimum inhibition concentration (MIC) of tested samples, the agar plate method was used; two-fold serial dilutions of each sample were added to nutrient broth for bacteria (beef extract 5 g, peptone 10 g added to 1000 mL distilled water, pH 7.0) and to sabourad dextrose broth for fungi, DMSO was used as the control. Then they were heated in an autoclave at 121 °C for 25 min. The culture of each organism was diluted by sterile distilled water to 105–106 CFU/mL, a loop of each suspension was inoculation, the plates were incubated at 37 °C for 24 h for bacteria, and at 30 °C for 3–4 days for fungi. The colonies were counted and the MIC values were obtained. The MIC was considered to be the lowest concentration that completely inhibits against inoculums compared with the control, disregarding a single colony or a faint haze caused by the inoculums [36].

## 4. Conclusions

Four novel hydrogels based on chitosan were successfully prepared via a cross-linking reaction between chitosan and various amounts of oxalyl bis 4-(2,5-dioxo-2*H*-pyrrol-1(5*H*)-yl)benzamide. Their structures have been confirmed using FTIR spectra, SEM and X-ray diffraction. All the hydrogels, compared with chitosan, have a higher antimicrobial activity as judged by their higher inhibition zone diameters and their lower MIC values. The results show that the hydrogels have a stronger activity against Gram-positive bacteria than against Gram-negative bacteria. These hydrogels also show a significant inhibitory effect on the fungi. The hydrogel of the lowest degree of cross-linking has a noticeably higher antibacterial and antifungal activity than those of the other hydrogels. Thus, it can be concluded that these hydrogels have an interesting usage as antimicrobial substances.

## Figures and Tables

**Table 1:** Swell ability (%) for the hydrogels in various solvents.

Samples	Swell ability (%)				

	Acetic acid solution (1% *v*/*v*)	DMSO	NMP	DMF	Distilled H_2_O
H_0.5_	10217	973	550	536	750
H_1_	1959	762	528	487	572
H_2.5_	1509	685	457	423	561
H_5_	988	626	385	327	500

**Table 2:** Inhibition indices of chitosan and its hydrogels against *B. subtilis*, *S. aureus*, *S. pneumonia*, *E. coli* and *S. typhimurium*.

Inhibition zone (mm) Tested microorganisms					

	Gram positive bacteria			Gram negative bacteria	
		
Samples	*B. subtilis* (RCMBA 6005)	*S. aureus* (RCMBA 2004)	*S. pneumonia* (RCMB 000101)	*E. coli* (RCMBA 5003)	*S. typhimurium* (RCMB 000104)
Chitosan	16.4	14.1	12.7	8.3	12.6
H_0.5_	22.4	20.6	19.9	13.9	16.4
H_1_	18.3	16.7	17.8	12.1	14.3
H_2.5_	17.5	15.9	14.6	12.0	13.9
H_5_	16.9	15.1	13.1	10.3	12.9

**Table 3:** Minimum inhibitory concentration (MIC) values of the hydrogels against *B. subtilis*, *S. aureus*, *S. pneumonia*, *E. coli* and *S. typhimurium*.

Minimum inhibitory concentration ( μg/mL) (MIC)					

	Gram positive bacteria			Gram negative bacteria	
		
Samples	*B. subtilis* (RCMBA 6005)	*S. aureus* (RCMBA 2004)	*S. pneumonia* (RCMB 000101)	*E. coli* (RCMBA 5003)	*S. typhimurium* (RCMB 000104)
Chitosan	62.5	125	125	500	250
H_0.5_	0.98	3.91	3.91	125	62.5

**Table 4:** Inhibition indices of chitosan and its hydrogels against *A. fumigatus* and *A. niger*.

Inhibition zone (mm) Tested microorganisms		

Samples	*A. fumigates* (RCMBA 06002)	*A. niger* (RCMBA 06106)
Chitosan	11.7	13.6
H_0.5_	18.7	20.1
H_1_	15.2	17.3
H_2.5_	15.9	15.4
H_5_	12.4	13.9

**Table 5:** MIC values of the hydrogels against *A. fumigatus*, and *A. niger*.

Minimum inhibitory concentration (MIC) (μg/mL)		

Samples	*A. fumigates* (RCMBA 06002)	*A. niger* (RCMBA 06106)
Chitosan	250	125
H_0.5_	15.63	3.91
